# Surgical Ventricular Entry is a Key Risk Factor for Leptomeningeal Metastasis of High Grade Gliomas

**DOI:** 10.1038/srep17758

**Published:** 2015-12-04

**Authors:** Roland Roelz, Peter Reinacher, Ramazan Jabbarli, Rainer Kraeutle, Beate Hippchen, Karl Egger, Astrid Weyerbrock, Marcia Machein

**Affiliations:** 1 Department of Neurosurgery, University Medical Center Freiburg, Breisacher Str. 64, 79106 Freiburg, Germany; 2 Department of Stereotactic and Functional Neurosurgery, University Medical Center Freiburg, Breisacher Str. 64, 79106 Freiburg; 3 Department of Neurosurgery, University Hospital Essen, Hufelandstr. 55, 45147, Germany; 4 Department of Nursing-IT, University Medical Center Freiburg, Hugstetter Str. 55, 79106 Freiburg; 5 Department of Neuroradiology, University Medical Center Freiburg, Breisacher Str. 64, 79106 Freiburg, Germany

## Abstract

Leptomeningeal metastasis (LM) of high grade gliomas (HGG) can lead to devastating disease courses. Understanding of risk factors for LM is important to identify patients at risk. We reviewed patient records and magnetic resonance imaging (MRI) of all patients with a first diagnosis of HGG who underwent surgery in our institution between 2008 and 2012. To assess the influence of potential risk factors for LM and the impact of LM on survival multivariate statistics were performed. 239 patients with a diagnosis of HGG and at least 6 months of MRI and clinical follow-up were included. LM occurred in 27 (11%) patients and was symptomatic in 17 (65%). A strong correlation of surgical entry to the ventricle and LM was found (HR: 8.1). Ventricular entry was documented in 137 patients (57%) and LM ensued in 25 (18%) of these. Only two (2%) of 102 patients without ventricular entry developed LM. Median overall survival of patients after diagnosis of LM (239 days) was significantly shorter compared to patients without LM (626 days). LM is a frequent complication in the course of disease of HGG and is associated with poor survival. Surgical entry to the ventricle is a key risk factor for LM.

While manifestation of malignant gliomas outside the central nervous system is extremely rare^1^,^2^, leptomeningeal metastasis (LM) is a well-known but widely underestimated complication in the course of disease of these patients. To date, we are unaware of the causes of LM and methods and techniques of prevention are not available. Although frequently detected in the absence of clinical symptoms LM can be the source of massive morbidity and poor survival of patients after diagnosis of LM has unequivocally been reported^3^,^4^,^5^,^6^,^7^,^8^,^9^.

Our knowledge about LM is based on small retrospective case series and case reports and its incidence and risk factors are incompletely understood. As multimodal glioblastoma treatment including surgery and chemoradiation extends overall survival from less than one year to up to 26 months, the likelihood of observing multifocal tumor dissemination might increase in the late course of the disease. While surgery, especially including ventricular entry, has been suspected to be associated with LM by some authors^3^,^8^,^10^,^11^ this notion has been challenged by others^4^.

Based on the largest patient cohort with long-term MRI follow-up investigated with respect to LM to date, the main objective of this study was to establish the incidence and risk factors of LM in patients with malignant gliomas. In particular, we intended to clarify the role of surgical opening of the ventricles for LM. Furthermore, the impact of LM and ventricular entry on patient survival was analyzed.

## Materials and Methods

The study design and methods were approved by the Ethics Committee of the University Medical Center Freiburg (reference #516/13). The methods were carried out in accordance with the approved guidelines. All persons or their relatives gave their informed consent within written treatment contract on admission and therefore prior to their inclusion in the study.

The study is reported based on criteria from the STROBE (Strengthening the Reporting of Observational Studies in Epidemiology) statement^12^.

399 patients with a first diagnosis of unifocal malignant glioma (WHO-grade III: anaplastic astrocytoma, anaplastic oligodendroglioma, anaplastic oligoastrocytoma; WHO-grade IV: glioblastoma and gliosarcoma) underwent surgical resection or biopsy in the Department of Neurosurgery at the University of Freiburg between 2008 and 2012. Patients with primary multifocal gliomas were excluded. 239 patients had at least 6 months of MRI and clinical follow-up. They were included in this study and their imaging studies and medical records were retrospectively analyzed. Pre- and postoperative and all follow-up MRI’s were reviewed for leptomeningeal metastasis, tumor size (defined as maximum tumor diameter in either axial or sagittal or coronal plane), proximity of the tumor to the ventricle (defined as minimum tumor distance to the border of any ventricle in either axial or sagittal or coronal plane) and surgical entry to the ventricle. Contiguity of the tumor to the ventricle was defined as non-measurable distance of contrast enhancing tumor to the ventricular wall. Ventricular entry was defined radiologically based on postoperative MRI and these findings were correlated to surgical reports.

In patients receiving only a stereotactic biopsy, the imaging documentation of the biopsy trajectories were reviewed for a transventricular course.

Leptomeningeal metastasis (LM) was defined as presence of an ependymal/subependymal or extraventricular subarachnoid/dural tumor manifestation distant from the primary tumor (absence of T1w hypointensity, T2w or FLAIR hyperintensity or Gadolinium-enhancement bridging both lesions). This was clearly differentiated from potential other sources of secondary tumor manifestations not related to CSF seeding: Subependymal tumor spread per continuitatem and multifocal tumor progression (defined as emergence of T2w/FLAIRw hyperintense lesions on follow-up MRI not in contact with the primary lesion or new contrast enhancing lesions that are not bridged to the primary tumor by T2w/FLAIRw hyperintensities) were not interpreted as LM.

### Follow-up Evaluation and Statistical Analysis

For survival analyses, patients were followed until death or censored at the end of follow-up (May 30, 2014). Patients lost to follow-up were censored at the last date known alive. To assess crude associations of variables univariate statistics were performed by log-rank test. For log rank tests, continuous variables were either dichotomized (distance of tumor to ventricle into contiguous and non-contiguous) or categorized in four categories of balanced sizes (age, tumor size). Categorical variables were described with proportions and percentages.

Two multivariable cox-regression analyses were set up. In the first model, the occurrence of **LM** was the selected failure event. The variables age, sex, tumor size, tumor location, distance of tumor to the ventricle, ventricular entry during surgery, WHO-grade, extent of resection and the number of tumor resections were evaluated for the effect on the occurrence of LM.

In the second model, we evaluated the impact of LM and the above mentioned parameters plus parameters of adjuvant therapy (radiotherapy, 2^nd^ radiotherapy at recurrence, temozolomide chemotherapy, PC/PCV chemotherapy and bevacizumab therapy) on survival and selected **death** as statistical failure event. To avoid an a-posteriori analysis regarding the impact of the variables ventricular entry and LM, they were analyzed as time-dependent variables. Both variables carry the baseline value of 0 that changes to 1 only at the time point of its occurrence. This may be at the time of diagnosis or any later date through-out the follow-up. In Stata13 this was achieved by splitting the dataset at event times. For ventricular entry the proportional hazard assumption was not met. To solve this problem an interaction with time was introduced. This lead to two different hazard ratios for ventricular entry (before and after 700 days). The proportional hazards assumption was verified using “Schoenfeld Residuals”. The effect of significant variables in the multivariable model was quantified using hazard ratios (HR). All reported p-values were two sided, and p < 0.05 was considered statistically significant. All statistical analyses were performed using Stata 13 statistical software (StataCorp. 2013. *Stata Statistical Software: Release 13*. College Station, TX, USA).

## Results

### Patient characteristics

Leptomeningeal metastasis was detected on MRI imaging of 27 (11%) of 239 consecutive patients with a first diagnosis of malignant glioma and surgery or biopsy between 2008 and 2012 at our institution. A comparison of patient groups with and without LM regarding patient-, tumor- and surgery-related characteristics and the corresponding univariate and multivariate statistics are summarized in Table 1.

Patient age did not differ significantly between groups (56 vs. 58 years). There was a tendency towards male sex (female to male ratio 1:2 vs. 1:1.3). However, these differences did not reach statistical significance. At the time of data analysis, median period of clinical and imaging follow up was insignificantly longer (498 vs. 585 days) for patients without LM. Patients with LM were more likely to be dead (96% vs. 68%) by the end of the observation period. 9 patients without LM on the last MRI were lost to follow-up. Statistical significance of the survival difference was maintained regardless of whether these 9 patients were excluded or considered dead or alive for statistical analysis. They were censored at their last known follow-up date.

### Tumor characteristics

**Figure 1:**
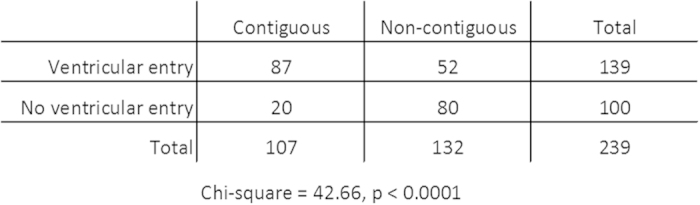
Contingency table showing the correlation between contiguity of tumor to the ventricle and ventricular entry during surgery. 72% of patients with a tumor contiguous to the ventricle had surgical ventricular entry. In contrast, only 39% of patients with a non-contiguous tumor had surgical ventricular entry.

There was no apparent difference in histology at diagnosis between groups. Primary histology in patients with LM was WHO-Grade IV in 22 (81%) and WHO-Grade III in 5 (19%) compared to 163 (77%) WHO-Grade IV tumors (157 GBM and 6 gliosarcomas) and 49 (23%) WHO-Grade III tumors in patients without LM. Median maximum tumor diameter was significantly larger 54.5 (range 18–96 mm) in LM patients compared to 42 (range 8–88 mm) in patients without LM. All tumors were classified as either contiguous or non-contiguous to any border of the cerebral ventricles. Significantly more tumors (19 of 27, 78%) in patients who later developed LM were contiguous to the ventricle compared to tumors of patients without LM (88 of 212, 42%). However, a causal relationship between contiguity of the primary tumor to the ventricle and LM could not be established. As expected, ventricular opening occurred more frequently in patients with tumors located in the vicinity of the ventricle as shown in Fig. 1. The tumor was contiguous to the ventricle in 107 patients and 87 (81%) of these had surgical entry to the ventricle. In contrast, 132 patients had a non-contiguous tumor and only 52 (39%) of these had ventricular entry. In the multivariate model contiguity of the tumor to the ventricle was eliminated as an independent risk factor for LM. There was no significant difference with regard to primary tumor location between groups. A true multifocal tumor progression – defined as intraparenchymatous emergence (on follow-up MRI) of T2w/FLAIRw hyperintense lesions not in contact with the primary lesion or new contrast enhancing lesions that are not bridged to the primary tumor by T2w/FLAIRw hyperintensities – was detected in 14 patients (6%). Multifocal progression occurred in 1 patient with LM (4%) and 13 patients without LM (6%).

At the time of diagnosis of LM the primary tumor was in contact with the ventricle in 100% of cases and considered progressive (e.g. contrast enhancing) in 21 patients (78%).

### Surgical characteristics

Both groups were nearly identical with regard to the number of tumor surgeries and the extent of resection (EOR). In total, 33 biopsy cases without open resection were included in this study. Two of these (7%) later developed LM. 52% of patients with LM compared to 44% of patients without LM were operated once. Two resections were performed in 22% (LM) and 30% (no LM) of patients, respectively. 19% (LM) and 11% (no LM) of patients had more than two tumor surgeries.

A gross total resection (GTR, >90% tumor reduction) was achieved during the first surgery in 70% of patients with LM and 68% of patients without LM. 22% and 17%, respectively, had a subtotal resection (STR, 50–90%). There were no partial resections (PR, <50%). 2 (7%) of patients who later developed LM had only a biopsy. 1 of 33 biopsies was performed via a transventricular trajectory. LM occurred in this patient after 316 days.

**Figure 2:**
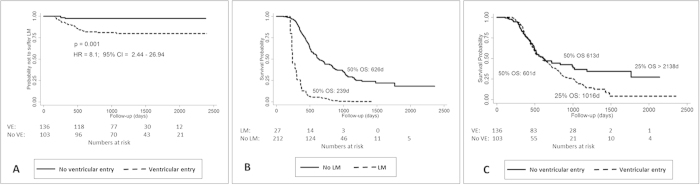
(**A**) Kaplan-Meier survival estimates (Event: LM) for the influence of ventricular entry on LM. Ventricular entry confers a high risk for LM (HR: 8.1). (**B**) Kaplan-Meier survival estimates of patients with LM (analyzed as time-dependent variable) compared to patients without LM. Occurrence of LM confers significantly shorter overall survival. (**C**) Kaplan-Meier survival estimates of the study population for surgical ventricular entry. Beyond 700 days of follow-up, ventricular entry has a negative effect on overall survival.

The first multivariable model with LM being the failure event (Fig. 2A) revealed a single and statistically highly significant effect for ventricular entry (p = 0.001). A hazards ratio of 8.1 (95% CI = 2.44–26.94) for ventricular entry was determined. The ventricle was entered in 25 of 27 patients (i.e. 100% of patients with open resection, not biopsy) who later developed LM. In contrast, the ventricle was entered in 112 of 181 surgical patients (62%) without LM. In both groups ventricular entry occurred during the first surgery in the majority of patients (88 vs. 76%).

The impact of ventricular entry on LM became even more apparent when patients were analyzed in a dichotomized fashion for presence or absence of ventricular entry. Overall, surgical entry to the ventricle was documented in 137 patients (57%) and CSF-dissemination ensued in 25 (18%) of these. Only two (2%) of 102 patients without ventricular entry developed LM.

### Clinical features of LM

#### Location, timing and symptoms of LM

**Figure 3:**
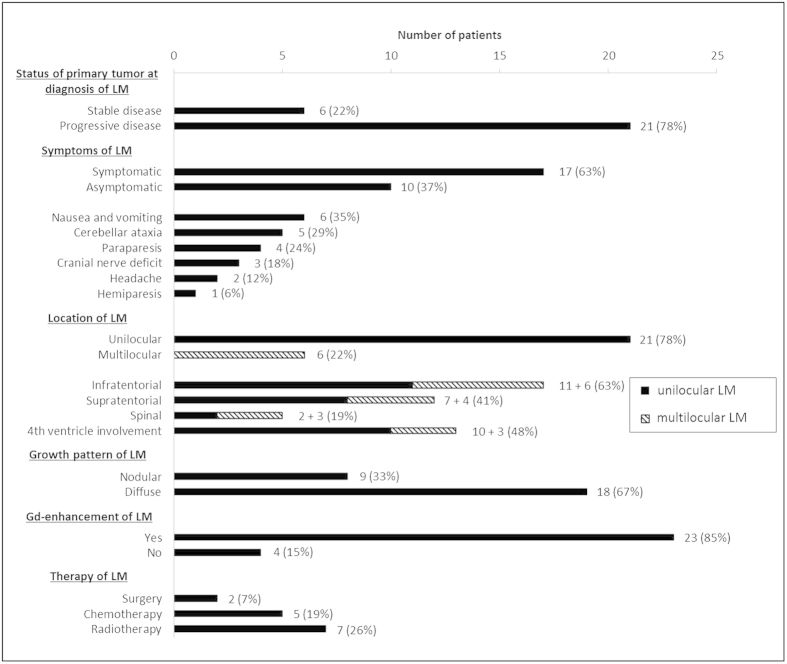
Clinical Presentation, MRI Features and Therapy of 27 Patients with LM.

Clinical features of LM are summarized in Fig. 3. At the time of diagnosis of LM, the primary tumor was judged to be in the state of progressive disease (PD) in 21 (78%) and stable disease (SD) in 6 of 27 patients (22%). LM was symptomatic in 17 patients (63%) and an asymptomatic imaging finding in 10 patients (37%). The most common symptoms were attributed to manifestation of LM in the posterior fossa: 6 patients (22%) suffered from nausea and vomiting and 5 patients (19%) had cerebellar ataxia. 4 patients (15%) presented with paraparesis due to spinal LM and 3 patients (11%) had cranial nerve deficits. Headache attributable to LM was the leading symptom in 2 patients (7%) and LM caused hemiparesis in 1 patient (4%).

LM was unilocular in 21 patients (81%) and multilocular in 6 patients (23%). The most common location of LM was infratentorial. 11 patients had isolated infratentorial LM. An infratentorial metastatic lesion occurred in 6 patients with multilocular LM. 48% of the patients with LM displayed a lesion in the fourth ventricle making this anatomic space the predilection site for LM.

The median time between the operation with ventricular entry and diagnosis of LM was 315 days (range 67–1070 days). The median time between initial diagnosis of HGG and LM was 377 days (range 67–1070 days).

#### Imaging features of LM

**Figure 4:**
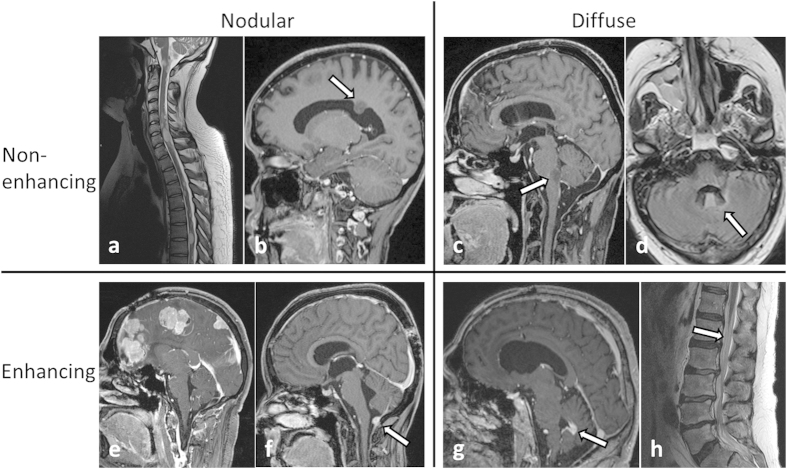
MRI examples of LM in the study population (a–c and e–h: T1w +Gd, d: T2-FLAIRw MRI). Two general morphological types of LM – nodular (**a+b, e+f**) or diffuse (**c+d, g+h**) – were observed. Both types occurred as enhancing (lower row) or non-enhancing (upper row) lesions. White arrows indicate sites of LM.

Morphologically, the cases of LM in the present cohort could be divided into two groups according to the growth pattern of LM. LM occurred as nodular lesion(s) in 9 patients (33%). A diffuse growth pattern was observed in 18 patients (67%). Both growth patterns occurred in a mutually exclusive fashion, i.e. presence of both nodular and diffuse growth of LM in one patient was not observed. Metastatic lesions were gadolinium-enhancing in 23 patients (85%) and non-enhancing in 4 patients (15%). Again, presence of both enhancing and non-enhancing lesions in a single patient was not observed. Figure 4 shows two examples for each type of nodular non-enhancing (a + b), diffuse non-enhancing (b + c), nodular enhancing (d + e) and diffuse enhancing (f + g) LM.

#### Therapy of LM

A specific therapeutic consequence from the diagnosis of LM was drawn in only 9 (33%) of 27 patients affected. This was due to the fact that most cases of LM were diagnosed in the late stage of the disease where best supportive care was deemed the most appropriate treatment choice.

Surgery for LM was performed in two patients. One patient with spinal LM and severe paraparesis underwent emergent decompressive surgery followed by radiotherapy. The patients motor functions improved slightly but she remained unable to walk. However, she regained autonomic functions. She required insertion of a ventriculo-peritoneal shunt several weeks later and survived for a period of 10 months after diagnosis of LM. The second patient underwent elective resection of a nodular manifestation of LM in the fourth ventricle and received adjuvant chemotherapy. He was not symptomatic at diagnosis of LM and recovered from surgery without additional deficits. There were no signs of recurrence under bevacizumab therapy 3 months after surgery.

The diagnosis of LM led to a change or reinstitution of chemotherapy in two patients. They survived 3 and 6 months, respectively. Metastatic lesions were irradiated in three patients with a survival of 3, 8 and 9 months after diagnosis of LM. 2 patients received both radiotherapy and chemotherapy and one of them died after 1 month. The other patient with combined treatment was alive and without progression at the end of observation period 15 months after initiation of therapy.

#### Survival Analysis: Shorter Overall Survival in Patients with LM

To investigate the impact of LM on survival the second multivariable model was fit to the failure event “death” (Table 2). LM was introduced as a time-dependent co-variable and the variables age, sex, tumor size, distance of tumor to the ventricle, ventricular entry during surgery (time-dependent), WHO-grade and the number of tumor resections remained within the model. In addition, variables of adjuvant oncological therapy (radiotherapy, 2^nd^ radiotherapy at recurrence, temozolomide, PC/PCV chemotherapy and bevacizumab therapy) were included. Higher **age at diagnosis (HR 1.03 per year)**, higher **WHO-Grade (HR 2.17 for grade IV)**, **LM (HR 3.62)** and lower **extent of resection (HR 1.78 for subtotal resection, HR 3.21 for biopsy)** had a significant negative influence on overall survival. Median OS of patients with LM was 239 days compared to 626 days in patients without LM (Fig. 2B).

The influence of ventricular entry is only seen after 700 days (see separation of curves on Kaplan Meier Chart, Fig. 2C, after 700 days). Median OS with and without ventricular entry is almost identical, whereas the 25% OS is >2138 days without ventricular entry and only 1016 days with ventricular entry.

## Discussion

In this study we determined the MRI-based incidence and the clinical characteristics of LM in 239 consecutive surgically treated patients with HGG. We found that ventricular entry during surgery is the key risk factor for LM. Patients who develop LM in the course of disease have an inferior survival compared to patients without LM. Ventricular entry is associated with shorter OS only seen after 700 days.

Primary central nervous system malignancies with a well-known propensity for leptomeningeal dissemination include predominantly pediatric tumors like medulloblastoma, pineoblastoma and ependymoma and surveillance neuroaxis screening by MRI is established for these tumors^13^. LM of a malignant glioma was first described by Cairns *et al.* in 1931^14^. Today, our understanding of this complication is still incomplete and based on a small number of studies and case reports.

The fact that LM is not uncommon in patients with HGG was established by early autopsy series documenting an incidence for spinal LM of 20–30%^15^,^16^. In contrast, symptomatic spinal dissemination was found to be rare ranging from 1 to 2.7%^3^,^17^,^18^. This discrepancy highlights the risk for an increasing number of patients with symptomatic LM in the future as improvement in local tumor control by advances in surgical, radiotherapeutic and chemotherapeutic management is beginning to pay off in prolonged patient survival^19^,^20^.

In line with recent MRI-based series^8^,^11^ we found a high LM incidence of 11% in patients with HGG with two thirds of these being symptomatic. MRI is today’s method of choice for the detection of LM. We did not include cytological analysis of CSF samples as a variable in this study since the number of false-negative results is known to be high^7^,^10^,^21^,^22^,^23^.

The 4^th^ ventricle is clearly the site of predilection for LM. Half of our patients with LM suffered from typical and disabling symptoms of cerebellar or rhomboid fossa lesions. Fujimura *et al.* and Cohen *et al.* have previously stressed the clinical relevance of LM in this location^24^,^25^. Particular attention should therefore be given to the 4^th^ ventricle in any follow-up MRI of patients with HGG. Neurooncologists should bear in mind that nausea and vomiting might be due to 4^th^ ventricle LM and not secondary to chemotherapy, hydrocephalus or increased intracranial pressure from tumor edema. CSF dynamics are the most likely explanation for the high frequency of LM in the 4^th^ ventricle. As demonstrated by phase contrast cine MRI studies investigating CSF dynamics the 4^th^ ventricle serves as a mixing chamber for CSF. While CSF flow is unidirectional in most CSF spaces it is multidirectional in the 4^th^ ventricle presumably leading to a prolonged stay of CSF within the 4^th^ ventricle^26^. We hypothesize that this particularity of CSF flow facilitates attachment of disseminated tumor cells to the ventricular wall with subsequent invasion and tumor growth.

Differential growth patterns of LM have been described earlier by several authors^11^,^15^. In basic accordance with these reports we observed two general types of LM: LM occurred as nodular lesion(s) in 33% or as diffuse lesion(s) in 67% of LM cases. Contrary to Bae *et al.*^11^ we did not detect mixed appearance of both types of LM in one patient. Onda *et al.*^15^ previously reported that invasive LM was more likely to be symptomatic. We did not find differences with regard to symptoms and survival between patients with infiltrative and nodular LM.

### Clinical risk factors

Several authors have speculated about an association of LM with young age^7^,^15^,^16^,^27^,^28^. However, we and others could not confirm this connection^11^. Although statistically not significant, considerably more male (17) than female (10) patients were affected by LM in our series. Since this tendency was very strong we hypothesize that, with greater patient numbers, male sex could almost certainly be established as an independent risk factor. This correlation was also described by others^10^. Grabb *et al.* found an association between LM and multiple resections and oligodendroglial differentiation in a pediatric cohort^10^ and multiple surgeries have recently been suspected to cause LM in patients with low-grade glioma^29^. WHO-Grade and tumor histology as well as the number of tumor surgeries had no influence on the probability of LM in our patients. In accordance with the pertinent literature, the particular supratentorial location was irrelevant for LM in our series. Strikingly however, both patients with a cerebellar location of the primary tumor later developed LM. The small case numbers are inadequate to permit a statistical analysis but HGG of the posterior fossa in adults appear to be associated with a more aggressive and invasive growth pattern and a higher risk for LM. Salazar *et al.* reported a 60% “seeding”-rate in posterior fossa GBM and later noted LM in 5 of 6 children with cerebellar HGG. He consequently suggested spinal irradiation for these rare tumors in the pediatric population^30^. Tsung *et al.* found LM in 4 of 21 cerebellar GBM and Endo *et al.* discovered LM in all 10 cases (of which 5 were operated) of cerebellar HGG^31^,^32^.

Recently, the subventricular zone (SVZ), a neurogenic niche in the adult brain lining the wall of the lateral ventricles has captured the attention of neuro-oncologists. A more aggressive and invasive behavior of malignant gliomas in contact with the SVZ and inferior survival of patients with such tumors were reported. It has been hypothesized that brain tumor stem cells contributing to HGG initiation, spread and recurrence originate from the SVZ and therefore inclusion of the SVZ into the radiation field would confer a superior prognosis^33^,^34^,^35^. These findings have been challenged by others and the true role of the SVZ for malignant gliomas is the subject of an ongoing animated discussion^36^,^37^. While ventricular entry is obviously more frequent in patients with tumors contiguous to the ventricle, our data imply that the periventricuar localization itself – and thus contact of the tumor to the SVZ - is not a risk factor for LM.

### The role of ventricular entry

The single and highly significant risk factor for LM was surgical opening of the ventricle. The risk for a patient with ventricular entry to experience LM in the course of disease is 8 times that of a patient without ventricular entry. This gives way to the question: Is LM a neurosurgical complication? LM may undoubtedly occur without previous surgery^30^,^38^,^39^,^40^ but studies specifically addressing the risks of ventricular entry are scarce. Elliot *et al.* did not find a correlation between ventricular entry and LM in their series of 51 HGG patients (of whom 35% developed LM). Bae *et al.* reported ventricular entry in 35 of 96 patients with HGG and all 11 patients with LM had previous ventricular entry. In line with these and our findings, several other authors found LM to be related to ventricular entry^3^,^8^,^10^,^16^. In light of the large variance of time between surgical ventricular entry and the diagnosis of LM observed in the present study (67–1070 days), non-surgical mechanisms like tumor recurrence with breach of the ventricles or local recurrence in the setting of a previously opened ventricle have to be taken into consideration as potential cause of LM.

In the 33 patients managed by biopsy only reported here, 2 still developed LM. It is unclear whether a transventricular biopsy carries a comparable risk for LM as surgical ventricular entry. In the patients included in this study a transventricular biopsy was performed in only one patient. Strikingly, this one later developed LM.

### Is There a Molecular Basis for Leptomeningeal Dissemination?

The fact that the timing of LM occurrence is highly variable in relation to surgery suggests that dissemination of glioma cells that later form metastatic lesions does not necessarily occur at the time of surgery. Undoubtedly, biological features of glioma cells influence their propensity to form LM. Only a very small number of studies have investigated the molecular characteristics of LM in HGG. In a series of 44 patients with HGG by Izumoto *et al.* *phosphatase and tensin homolog* (PTEN) mutations were found in 5 of 15 patients (33%) with LM compared to 2 of 29 patients (7%) without LM^41^. Accordingly, Kato *et al.* found 7 PTEN mutations in 13 patients with disseminated HGG and only 1 PTEN mutation in 15 patients without dissemination^42^. PTEN acts as a tumor suppressor gene in part by inhibiting cell migration^43^ and PTEN loss leads to disinhibition of extracellular matrix-dependent PI3K/Akt cell survival^44^. Loss of PTEN function therefore might enhance adhesion-independent cell growth and invasion necessary for development of LM. Of note, presence of PTEN mutations delineates a more aggressive glioma phenotype as inferior survival of patients with PTEN mutated HGG has been demonstrated^45^.

Korshunov *et al.* performed molecular profiling of 8 disseminated glioblastomas and detected gain at 1p36 in all of these tumors. As 1p36 loss is a frequent cytogenetic alteration in glial tumors, this genetic alteration cannot be considered a risk factor for LM to date^46^,^47^. Taken together, our knowledge of biological features that promote LM is minimal. Our study furnishes evidence for an important contribution of surgical ventricular entry to the development of LM. Identification of molecular risk factors for LM is important but no candidate genes with prognostic relevance or molecular subgroups have been identified so far due to lack of large case series^41^,^42^.

Undelayed surgical resection to the maximum possible extent is the first-line treatment of choice for most malignant gliomas. Many studies have demonstrated the essential role of a high EOR for patients with high- and low-grade gliomas and this approach has found its way into treatment guidelines for these tumors^48^,^49^,^50^,^51^,^52^,^53^,^54^,^55^,^56^. Frequently, radical glioma surgery is associated with surgical entry to the ventricle. The present study shows that ventricular entry is the key risk factor for LM in patients with HGG. Furthermore, ventricular entry is associated with shorter OS given an appropriate length of follow-up (>700 days). Due to the preeminent significance of radical surgery we are convinced that ventricular entry should never be avoided at the cost of an incomplete resection. However, neurosurgeons must be aware that LM may be associated with this approach. Special attention to symptoms of LM like radicular, myelopathic or posterior fossa symptoms and a scrutinizing review of all MRI for distant lesions is warranted in follow-up examinations. As spinal LM is very rare routine neuroaxis screening is not warranted in HGG patients.

### Limitations of the study

Our study is subject to the common constraints of a retrospective investigation. We limited the study to patients with a follow-up of at least 6 months as LM represents a complication that usually occurs late after diagnosis and LM present at initial diagnosis is extremely rare in HGG^21^.

Because of the small number of patients with LM in the present cohort the study is underpowered to prove the significance of relevant risk factors other than ventricular entry (e.g.: young age, male sex) for LM.

Despite the clear result of the present study identifying ventricular entry during surgery as the key risk factor for LM, no conclusion can be drawn to the time point of the dissemination of tumor cells and to possible methods to prevent this. The highly variable interval (67–1070 days) between ventricular entry and occurrence of LM makes it unlikely that the tumor cell spread occurs during surgery unless one considers a long dormancy of tumor cells in the perivascular niche. There is no clear evidence if surgical methods to seal the ventricle wall or temporary external drainage can prevent LM. In addition to surgical aspects and the location of the tumor with reference to the ventricle, future studies have to focus on tumor-associated factors, e.g. the gene expression and epigenetic regulation of genes associated with invasion and metastasis which may determine the propensity of glioma cells to circulate in the CSF space and to cause tumor manifestations distant from the primary tumor mass.

## Additional Information

**How to cite this article**: Roelz, R. *et al.* Surgical Ventricular Entry is a Key Risk Factor for Leptomeningeal Metastasis of High Grade Gliomas. *Sci. Rep.* **5**, 17758; doi: 10.1038/srep17758 (2015).

## Figures and Tables

**Table 1:** Summary of clinical features of patients. Two multivariable statistical models were applied to assess the influence of these features on either LM (Failure Event: LM, Table 1) or overall survival (Failure Event: Death, Table 2).

Failure Event: Leptomeningeal Metastasis	Leptomeningeal Metastasis		Univariate Statistics (log rank)	Multivariate Statistics (Cox regression)
	Yes	No		
Patient characteristics				
Number of patients	27 (11%)	212 (89%)		
Sex			0.321	0.461
Male	18 (67%)	119 (56%)		
Female	9 (33%)	93 (44%)		
Age at diagnosis (y), median	56 (22–80)	58 (8–87)	0.301	0.431
Follow-up (d), median	498	585	0.193	
Status				
Dead	26 (96%)	145 (68%)	0.011	
Alive	1 (4%)	58 (27%)		
Unknown	0	9 (4%)		
Tumor Characteristics				
WHO-Grade			0.602	0.427
III	5 (19%)	49 (23%)		
AA	0	21 (10%)		
AOD	1 (4%)	12 (6%)		
AOA	4 (15%)	15 (7%)		
IV	22 (81%)	163 (77%)		
Glioblastoma	22 (81%)	157 (74%)		
Gliosarcoma	0	6 (3%)		
Tumor size (mm), median	54	42	**0.042**	0.917
Location of tumor				
Frontal	7 (26%)	71 (33%)	Reference for Location	
Temporal	13 (48%)	77 (36%)	0.314	0.854
Parietal	2 (7%)	34 (16%)	0.550	0.609
Other	5 (19%)	30 (14%)	0.397	0.815
Distance of tumor to ventricle			**0.006**	0.631
contiguous (0 mm)	19 (70%)	88 (42%)		
non-contiguous (>0 mm)	8 (30%)	124 (58%)		
Surgical Characteristics				
Number of tumor resections			0.663	0.352
0 (Biopsy)	2 (7%)	31 (15%)		
1	14 (52%)	93 (44%)		
2	6 (22%)	64 (30%)		
>2	5 (19%)	24 (11%)		
Extent of resection (1^st^ surgery)				
GTR (>90%)	19 (70%)	145 (68%)	Reference for Extent of Resection	
STR (50–90%)	6 (22%)	36 (17%)	0.686	0.956
PR (<50%)	0	0		
Biopsy	2 (7%)	31 (15%)	0.380	0.335
Ventricular entry§	25 (100%)	112 (62%)	**0.001**	**0.001** (CI 95%: 2.44–26.94) **HR: 8.1**
1^st^ surgery	22 (88%)	85 (76%)		
2^nd^ surgery	3 (12%)	25 (22%)		
>2^nd^ surgery	0	2 (2%)		
Adjuvant Therapy				
None	0	5 (2%)	1.000	
Radiotherapy	27 (100%)	190 (90%)	0.147	
2^nd^ Radiotherapy at recurrence	9 (33%)	47 (20%)	0.266	
Temozolomide	20 (74%)	149 (70%)	0.708	
PC/PCV*	5 (19%)	26 (12%)	0.432	
Bevacizumab	5 (19%)	34 (16%)	0.782	

^*^PC/PCV: Procarbacin and CCNU/Procarbazin, CCNU and Vincristin.

^§^Biopsy cases not included.

**Table 2:**
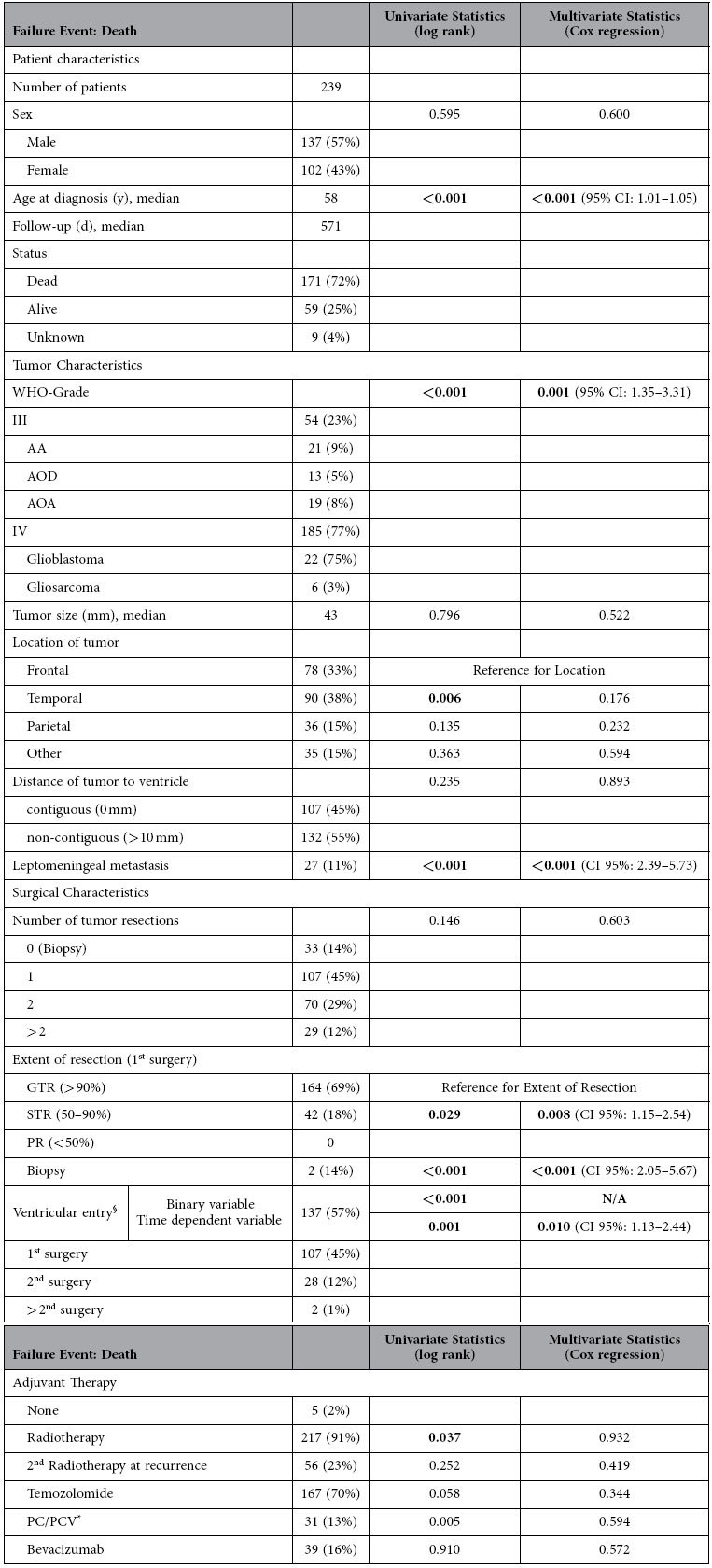
The only relevant risk factor for LM was surgical entry to the ventricle.

Older age at diagnosis, higher WHO-Grade, LM, lower extent of resection and ventricular entry (beyond 700 days of follow-up) were significant predictors of inferior overall survival.

N/A: Not applicable.

^*^PC/PCV: Procarbacin and CCNU/Procarbazin, CCNU and Vincristin.

^§^Biopsy cases not included.
